# Deficiency of SECTM1 impairs corneal wound healing in aging

**DOI:** 10.1111/acel.14247

**Published:** 2024-06-17

**Authors:** Jin Zhu, Xihong Lan, Kunlun Mo, Wang Zhang, Ying Huang, Jieying Tan, Li Wang, Jianping Ji, Qiong Ke, Hong Ouyang

**Affiliations:** ^1^ State Key Laboratory of Ophthalmology, Guangdong Provincial Key Laboratory of Ophthalmology Visual Science, Zhongshan Ophthalmic Center Sun Yat‐Sen University Guangzhou China; ^2^ Key Laboratory for Stem Cells and Tissue Engineering, Center for Stem Cell Biology and Tissue Engineering, Ministry of Education, Zhongshan School of Medicine Sun Yat‐Sen University Guangzhou Guangdong China; ^3^ Department of Histoembryology and Cell Biology, Zhongshan School of Medicine Sun Yat‐Sen University Guangzhou Guangdong China

**Keywords:** aging cell, corneal wound healing, limbal stem cell, SECTM1

## Abstract

The corneal epithelium is the outermost transparent barrier of the eyeball and undergoes continuous self‐renewal by limbal stem cells (LSCs) during its lifetime; however, the impact of aging on LSCs remains largely unknown. Here, we showed that the healing ability of the cornea in elderly macaques (*Macaca fascicularis*) was significantly decreased compared to that of younger macaques. This delayed wound closure accompanied a disordered cell arrangement and corneal opacity. A novel cytokine, Secreted and Transmembrane 1 (SECTM1), was found to facilitate corneal healing and was upregulated in young macaques upon wounding. Mechanistically, SECTM1 is essential for LSC migration and proliferation, and may partially function through Cell Division Cycle Associated 7 (CDCA7). Notably, the topical application of SECTM1 to aged wounded corneas dramatically promoted re‐epithelialization and improved corneal transparency in both mice and macaques. Our work suggests that aging may impair the expression of healing response factors and injury repair in non‐human primate corneas, and that SECTM1 application could potentially benefit corneal wound healing in clinical treatment.

## Graphical abstract

SECTM1 is upregulated in post‐wound cornea during healing process which is more pronounced in young individuals. It can promote healing speed by stimulating limbal stem cell proliferation and migration.

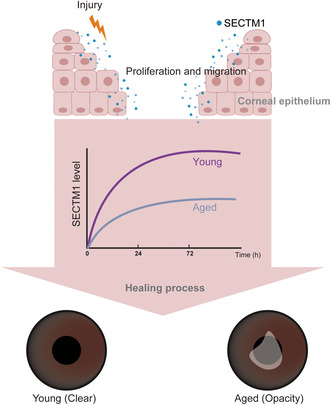

**Abbreviations**
CDCA7: Cell Division Cycle Associated 7
CSFE: carboxyfluorescein succinimidyl amino ester
GO: gene ontology
LSC: limbal stem cells
MGP: Matrix Gla Protein
SECTM1: Secreted and Transmembrane 1

## 1 INTRODUCTION

The frequency of age‐related issues has increased with time and is becoming a major risk factor for human health, causing functional alterations in organ tissues (Parrish, 2017). Mounting evidence indicates that aging can lead to a decrease in the efficiency of many biological events and a decline in homeostatic capacity (Hardman et al., 2005; Marcus et al., 2000). In particular, aging can decrease the self‐remodeling of organ epithelial tissue in the lungs (Schuliga et al., 2021) and intestine (Igarashi et al., 2019). During wound repair, aged skin tends to show impaired (Shook et al., 2018) and delayed healing (Blair et al., 2020). Thus, research to prevent the decline in function caused by aging is desirable.

The corneal epithelium is located on the ocular surface, and is extremely vulnerable to damage (Bashir et al., 2017). To maintain corneal transparency and refractive function, the corneal epithelium is constantly renewed and repaired by a population of unipotent limbal stem/progenitor cells (LSCs), which are believed to exist in the basal epithelium of the vascularized natural barrier between the cornea and conjunctiva (Cotsarelis et al., 1989; Davanger & Evensen., 1971; Gonzalez et al., 2018). Aging is responsible for structural and functional alterations in the corneal epithelium. A decline in the cell density of the basal layer (Zheng et al., 2016) and an increase in the mean cell diameter (Gambato et al., 2015) are observed in the corneal epithelium with increasing age. Furthermore, the limbal niche features are significantly reduced in the elderly (Notara et al., 2013). Age‐related changes, such as decreased corneal sensitivity (Roszkowska et al., 2004) and epithelial thickness deterioration (Yang & Hong, 2014), have also been reported. Although these studies demonstrated that senescent phenotypes increase with aging, the mechanism of aging in corneal wound healing remains largely unknown, impeding the prospects for therapeutic advances.

Corneal wound healing is a complex biological process involving immune responses and cytokines, followed by rapid proliferation and migration of corneal epithelial cells into the wound bed (Bukowiecki et al., 2017). Notably, delayed wound closure can lead to corneal scarring in which keratocytes are activated and irreversibly transformed into fibroblasts (Shu & Lovicu, 2017). Therefore, appropriate resurfacing of the corneal epithelium is essential for corneal transparency.

Here, we investigated the effects of aging on corneal wound healing in elderly versus young macaques (*Macaca fascicularis*). Delayed wound closure and corneal opacities have been observed in macaques. Secreted and Transmembrane 1 (SECTM1) which is upregulated only in the corneal epithelium of younger individuals after injury, influences LSC proliferation. Topical application of recombinant SECTM1 rescued the aging phenotype, promoted corneal wound healing, and reduced corneal opacity in monkeys and mice.

## 2 MATERIALS AND METHODS

### 2.1 Clinical materials

Human corneal tissues were obtained voluntarily from the donor in Eye Bank of Zhongshan Ophthalmic Center and approval by the Ethics Committee of Zhongshan Ophthalmic Center, Sun Yat‐sen University (2023KYPJ126).

### 2.2 Animal model and treatment

The study design and experimental protocols were approved by the Animal Ethics Committee of the Zhongshan Ophthalmic Center, Sun Yat‐Sen University (2018‐125 and P2023001). All animal‐related experimental procedures were done in accordance with NIH guide for the care and use of laboratory animals (https://www.ncbi.nlm.nih.gov/books/NBK54050/) and adhered to the Association Research in Vision and Ophthalmology. Young C57BL/6 mice (6–8 weeks old) were purchased from the Guangdong Provincial Centre (Guangzhou, China) and part of them were maintained as old mice (2 years) in the animal facility of Zhongshan Ophthalmic Center until 24 months. Young (2.2–4.7 years old) and aged (10–12.8 years old) *M*. *fascicularis* were purchased from Conghua Huazhen animal farm.

### 2.3 Subconjunctival injection in mice and macaques

After anesthetizing based on body weight, standard disinfection procedures were followed. The animal was positioned supine and fixed in place, with the eyes exposed using an eyelid retractor. Anesthesia of the ocular surface was achieved using hydrochloride bupivacaine eye drops, with excess fluid absorbed using a cotton swab. A corneal epithelial debridement tool was gently used to excise half of the temporal side limbus, corneal epithelium, and superficial stroma of both eyes. The macaques were injected subconjunctivally (at the 6 o'clock and 12 o'clock position) immediately after cornea scrape with 5μg/mL recombinant SECTM1 (R&D; 80 μL Recombinant Human SECTM1 per eye) five times (injection daily). The mice were anesthetized and injected subconjunctivally (at the 6 o'clock and 12 o'clock position) immediately after cornea scrape with 5μg/mL recombinant SECTM1A (R&D; 5 μL Recombinant Mouse SECTM1A per eye) three times (injection daily). The same volume of phosphate buffer saline (PBS) was injected as vehicle control.

### 2.4 Isolation and culture of human limbal stem cells

Cornea limbus tissue was obtained from donor and washed by cold PBS with 2% penicillin and streptomycin (P/S) and cut into small piece. Following 3 mg/mL collagenase IV digestion at 37°C for 1 h, the tissue clusters were obtained, and the digestion was terminated by adding equal volume of 10% FBS DMEM. Primary cells were seeded on cell culture plate coated with 1% Collagen I. Culture Medium for LSCs included DMEM/F12 (Gibco) and DMEM(Gibco) (1:1) with 1% penicillin/streptomycin (Gibco), 10 ng/mL EGF (Millipore), 10 μg/mL of insulin, 10^−9^ M 3,3′,5‐triiodo‐L‐thyronine, 0.2 μg/mL of hydrocortisone (Millipore), 0.1 nM cholera toxin, and 10% FBS. All reagents were purchased from Sigma–Aldrich, unless otherwise noted.

### 2.5 Corneal epithelial wound healing

Half of corneal epithelium was scraped with Algerbrush II corneal rust ring remover (Alger Co, Lago Vista, TX, USA).Corneal epithelium defects were visualized by instilling 0.25% fluorescein sodium and photographing them under a slit‐lamp microscope image system (SL‐D7/DC‐3/MAGENet; Topcon, Tokyo, Japan). The staining area was analyzed using ImageJ software (http://imagej.nih.gov/ij/; provided in the public domain by the National Institutes of Health, Bethesda, MD, USA) and calculated as the percentage of residual epithelial defects.

### 2.6 Evaluation of corneal regeneration

Corneal epithelial defects and opacity were photographed by slit lamp biomicroscope and assessed every day. To assess the epithelial defect, fluorescein‐stained regions of the corneal surface were measured using ImageJ (National Initiation of Health, NIH). Corneal opacity was scored as following scale: 0 = none, 1 = mild, 2 = moderate, 3 = severe, pupil seen faintly, and 4 = severe, pupil not visible (Bian et al., 2016). Success was defined as the scores <3 after treatment.

### 2.7 Immunofluorescence staining

Tissue immunofluorescence experiments was described as follows: Initially, the tissue specimens underwent a series of preparatory steps involving fixation, dehydration, paraffin embedding, and de‐paraffinization. Subsequently, the sections were treated for permeabilization and blocking utilizing by 0.3% Triton X‐100 and 3% bovine serum albumin for 1 h. Thereafter, incubated with primary antibodies overnight, followed by a secondary antibody incubation for 1 h. Nuclear staining was accomplished using DAPI. For immunocytochemistry (ICC), Limbal Stem Cells (LSCs) were cultivated under aseptic conditions on eight‐well glass slides (Millicell EZ‐Slide, EMD Millipore, Catalog #PEZGS0816). LSCs were cultured in sterile Millicell EZ‐Slide eight‐well glass plates (EMD Millipore, #PEZGS0816) and after indicated treatments, cells were fixed in 4% PFA for 15 min prior to immunostaining as described above for tissue immunofluorescence experiment. Images were acquired using a confocal fluorescence microscope (Zeiss, LSM 800). The antibodies mentioned in this section are listed in Table S1.

### 2.8 RNA interference and gene expression analysis

Two shRNAs targeting desired gene were respectively cloned into the PLKO.1 lentivirus plasmid. LSCs were infected with lentivirus particles encoding shRNAs for 16 h and then selected with 2 μg/mL puromycin (Gibco) for 48 h. Total RNA was extracted using the RNeasy Protect Mini Kit (Qiagen, #74124) and quantified with a spectrophotometer (NanoDrop ND‐1000; Thermo Fisher Scientific, USA). cDNAs were synthesized using the PrimeScript™ RT Master Mix Kit (Takara, #HRR036A). Gene expression was then measured by quantitative PCR (qPCR) using the iTaq™ Universal SYBR® Green Supermix Kit (Bio–Rad). The following shRNAs were used:

shSECTM1‐1: 5′‐TGTTCAAACCCTCACCACTT‐3′.

shSECTM1‐2: 5′‐CACCAGAGAAATAACAGACAA‐3′.

shCDCA7‐1: 5′‐AGAAACTCCAATCAAGTTAAT‐3′.

shCDCA7‐2: 5′‐GCGCTTATGTTACAAGTTGTT‐3′.

Scramble: 5′‐CAACAAGATGAAGAGCACCAA‐3′.

Primers are listed in Table S2.

### 2.9 Western blot

LSCs were collected after treatment. Then, cell was suspended by 100 μL RIPA lysis buffer supplemented with 1% protease inhibitor (Sigma–Aldrich, #P8340). For WB analysis, load equal amounts of the sample (10 μL) to perform electrophoresis. Then visualize using a suitable developing solution.

### 2.10 RNA‐seq data analysis

Alignment of RNA‐Seq reads to the reference genomes was accomplished using the STAR software (Dobin et al., 2013) to the human hg19 reference genome and monkey Macaca_fascicularis_5.0 reference genome. The calculation of Transcripts Per Million (TPM) values was conducted utilizing the RSEM tool (Li & Dewey, 2011). The time‐series dataset underwent a filtration process whereby genes exhibiting a consistent TPM value of 0 across all temporal data points were excluded. The identification of differentially expressed genes was carried out through the application of the DESeq2 R package (Love et al., 2014), with a log2 fold‐change value of ≥1 and a false‐discovery rate of <0.05. GO biological process enrichment analysis was performed using the clusterprofiler R package (Yu et al., 2012) with a *p*‐value cut‐off of 0.01 and a *q*‐value cutoff of 0.05. Heatmap was plotted by https://www.bioinformatics.com.cn (last accessed on 10 July 2023), an online platform for data analysis and visualization.

### 2.11 Wound healing assay and cell proliferation assay

Primary LSCs were seeded onto six‐well plates. When the cell confluence reached about 100%, scratch wounds were made by scraping the cell layer across each culture plate using the tip of 200 uL pipette. After wounding, the debris was removed by washing the cells with PBS. Wounded cultures were incubated in PBS, SECTM1 antibody or SECTM1 recombinant protein medium for 24 h, and then 3 fields (10X) were randomly picked from each scratch wound and visualized by microscopy to assess cell migration ability. Primary LSCs were labeled with CFSE (2 μM) and then treated with IgG, or with SECTM1 neutralizing antibody, or with SECTM1 recombinant protein. After 4 days in culture, the CFSE fluorescence was determined by flow cytometry and FlowJo V10 software.

### 2.12 Statistical analysis

All data are expressed as the mean ± SD or mean ± SEM. Statistical analyses were performed using GraphPad (GraphPad Software, USA). Student's *t*‐test was used to compare differences between groups. The criterion for statistical significance was *p* < 0.05.

## 3 RESULTS

### 3.1 Aged corneas experience longer re‐epithelization upon injury

**FIGURE 1:**
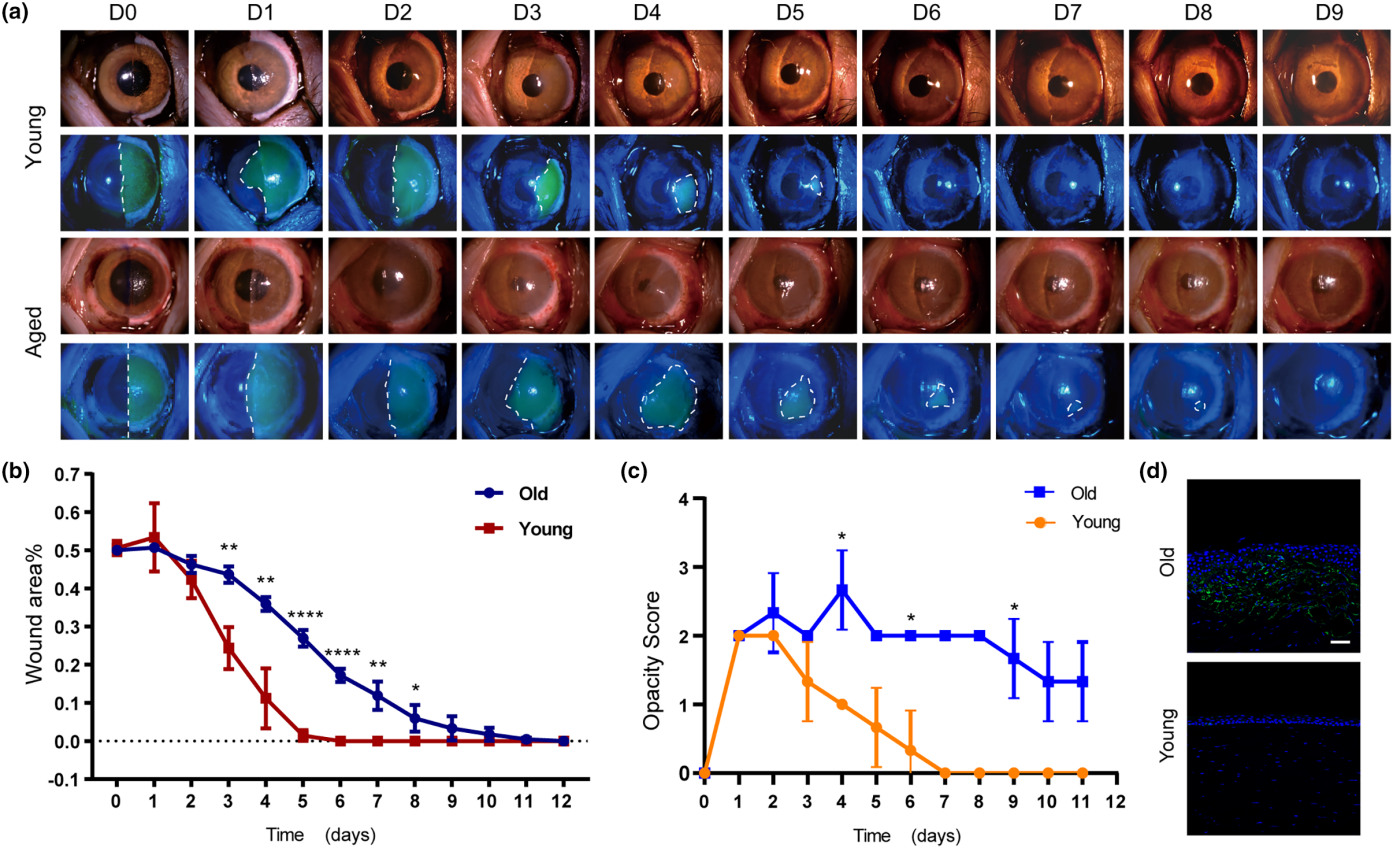
Corneal wound healing process in young and aged macaques. (a) Representative images of the temporal re‐epithelization process that occurs following cornea wounding (*t* = 0) in young and aged macaques. Slit‐lamp photographs of young and aged macaques wounded corneas at each time point (d0, d1, d2,…, d9 after the scrape of corneal epithelium). Fluorescence sodium dyeing was used to identify the area of epithelial defect. (b) Quantification of re‐epithelialization in young and aged wounds. Epithelial defect is presented as the percentage of the original wound size. Students *t*‐test was used to measure statistical significance, *n* = 3. Data are represented as means ± SD (*p < 0.05, **p < 0.01, ****p < 0.0001, compared with young group). (c) Corneal opacity is evaluated by opacity score. Kruskal–Wallis test was used to measure statistical significance, *n* ≥ 3. Data are represented as means ± SEM (*p < 0.05, compared with young group). (d) Immunofluorescent staining of alpha‐SMA on healed monkey corneas, scalebar =100um.

To explore differences in corneal wound healing between young and aged primates, we observed the repair process in young (1.5–2.5 years) and aged (12–15 years) *M. fascicularis*. The epithelium covering half of the corneal area was scraped and relatively fast sodium fluorescein staining was used to trace the healing process (Figure 1a,b, and Figure S1a). No significant differences were observed for the young and aged corneas in the first 2 days. However, beginning with Day 3, the younger corneas showed a robust repair speed compared with that of the aged corneas, and this was maintained consistently until the end of wound healing. At Day 4, the corneal wound area of the aged group had shrunk to 35.9 ± 1.5%, but it was still considerably greater than that of the young group, which had shrunk to 11.1 ± 6.4%. By Day 6, the corneal wound area in the aged group was still not completely closed (17.2 ± 1.4%), while young group had finished healing (Figure 1b). The cornea of aged group had to wait until Day 12 for completely healing. Notably, the younger group maintained corneal transparency after recovery, whereas the repaired corneas in the aged group became cloudy (Figure 1c). The opacity of aged cornea could be caused by the appearance of fibroblasts (α‐SMA^+^), which is absent in young cornea (Figure 1d).

Similar to the difference between the young and aged macaques, younger mouse corneal wounds healed in 3 days and remained transparent, whereas aged mouse corneal wounds did not close until Day 6 (Figure S1b–d). Collectively, the significant disparity between young and aged corneas during the repair process lies in the differences in healing time and visual quality.

### 3.2 Aged corneas display a less sensitive transcriptional response to wounding

Identifying the underlying mechanism responsible for delayed corneal wound healing caused by aging was the focal point of our investigation. We compared the RNA‐seq results of corneal tissues under homeostatic conditions (Control) with those of wound‐edge corneas to define a molecular signature footprint for the wound response. Pearson correlation analysis indicated that the gene expression of young and aged corneal epithelia post‐wounding could be distinguished from that of their control groups and each other (Figure S2a).

**FIGURE 2:**
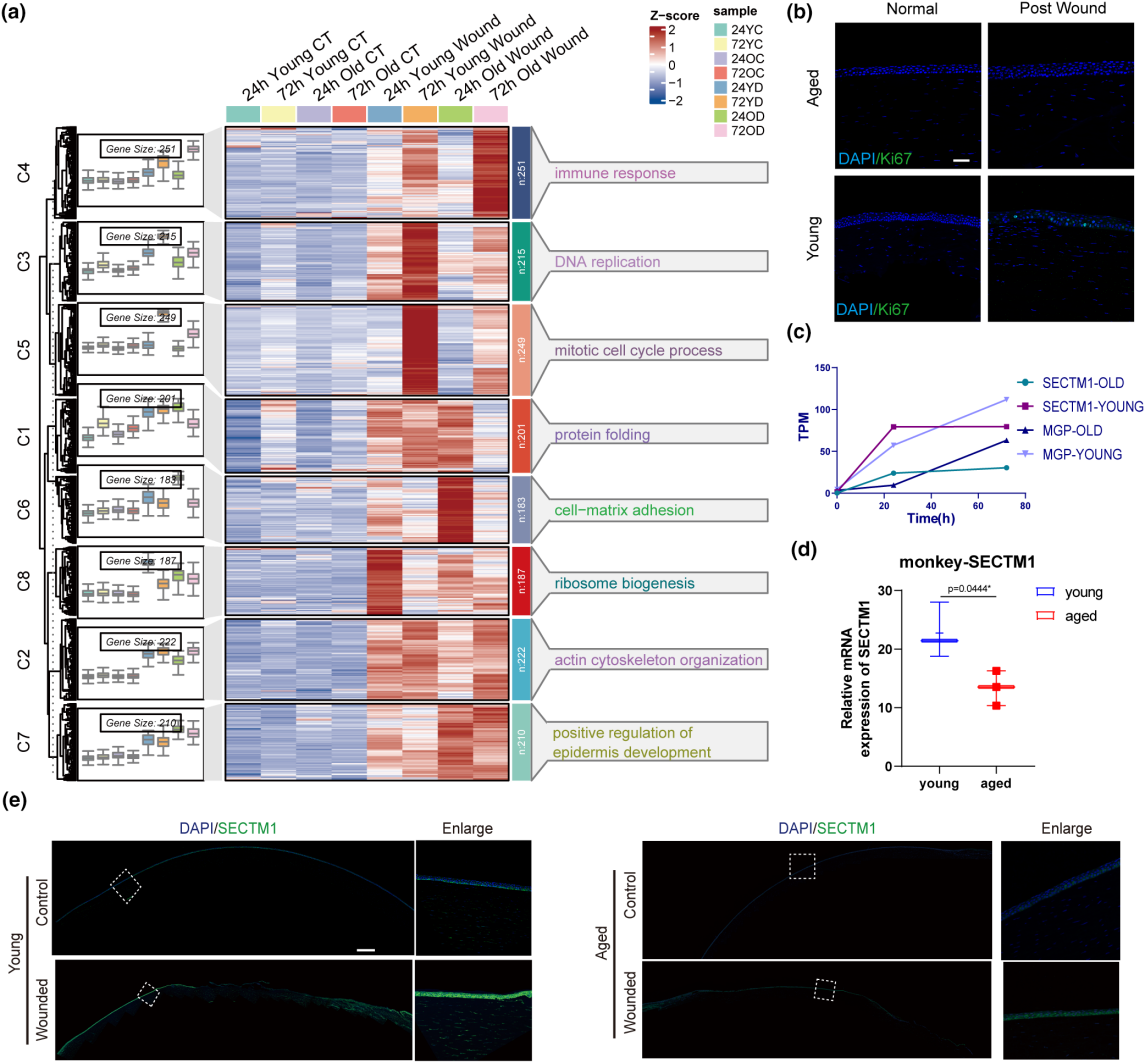
Gene transcriptional profile of injured corneal tissues in young and aged macaques. (a) Gene expression cluster analysis based on differential genes between young and aged post‐damage corneas and their uninjured control in different time points which represented by rows in heatmap. These differential genes were clustered by different variation tendency over time and tagged with their GO term. (b) Immunofluorescent staining of Ki67 on 24 h post‐wound monkey corneas, scalebar =100um. (c) SECTM1 and MGP gene expression trend between young and aged macaques corneas post‐damage over time which plotted by their TPM values. (d) SECTM1 mRNA expression upregulation level between young and aged macaques corneas post damage. q‐PCR values were normalized to the values of internal GAPDH. (e) Immunofluorescent staining of SECTM1 protein in 24 h post‐wound and unwound macaque corneas by corresponding young and aged conditions. Green signals represent SECTM1. Scalebar = 500um.

A heat map of the universal set of all upregulated genes was generated at each time point and compared to their respective controls, and clustering analysis was performed (Figure 2a). Interestingly, in the resting state (i.e., non‐injured), RNA transcriptome activation levels in both young and aged corneas exhibited minimal differences. Genes responsible for the observed expression variance accounted for only 0.125% (23 of 18,314) of the total genes (Figure S2g). During the process of injury repair, both young and aged corneas exhibited heightened activity in actin cytoskeleton organization (C2) and epidermal development (C7), suggesting that repair mechanisms were activated. Simultaneously, certain clusters (C4 and C6) appeared to be more pronounced in the elderly corneas, indicating that aged corneas prefer to enhance cell‐matrix and cell‐substrate adhesion at 24 h (Figure S2b) and the immune response at 72 h (Figure S2c).

Interestingly, the expression levels of certain genes in the C3 and C8 clusters at 24 h, were significantly higher in young corneas than in aged corneas immediately after injury. These genes were directly related to DNA replication (Figure S2e) and ribosome synthesis (Figure S2d), indicating substantial cell proliferation. To confirm this point, we stained the corneas in resting state and wound healing state with proliferation marker Ki67, and the increased Ki67 expression could only be observed in young corneas after wound healing in 24 h (Figure 2b). Accordingly, the expression of mitosis‐related genes (C5 cluster, Figure S2f) was observed in both young and aged corneas at 72 h; still, the intensity of expression in the aged corneas was notably lower, which is consistent with the delayed healing process (Figure 1).

We then focused on genes that responded early upon injury and maintained high‐level expression in young corneas, as this could help us identify key players in ensuring the superior regenerative capacity of young corneas. Therefore, genes within the C3 cluster tended to be favored for detailed investigation. In line with clinical application considerations, secreted proteins were the primary choice. Among the 215 genes expressed in the C3 cluster, two candidates were identified: *SECTM1* and *MGP* (Matrix Gla Protein). In particular, *SECTM1* exhibited significant wound‐induced upregulation and sustained expression in younger corneas, which was not observed in elderly corneas (Figure 2c).

SECTM1 is a transmembrane secreted protein with characteristics of a type 1a transmembrane protein. At 24 h post‐injury, the expression level of SECTM1 was significantly upregulated in the corneas of younger monkeys, whereas aged individuals showed a passivated response (Figure 2d). Consistently, immunofluorescence staining showed that only faint expression of SECTM1 was detected in unwounded corneas, regardless of age, and more pronounced SECTM1 expression was observed in young corneas upon wounding (Figure 2e).

### 3.3 SECTM1 is essential for LSC proliferation

**FIGURE 3:**
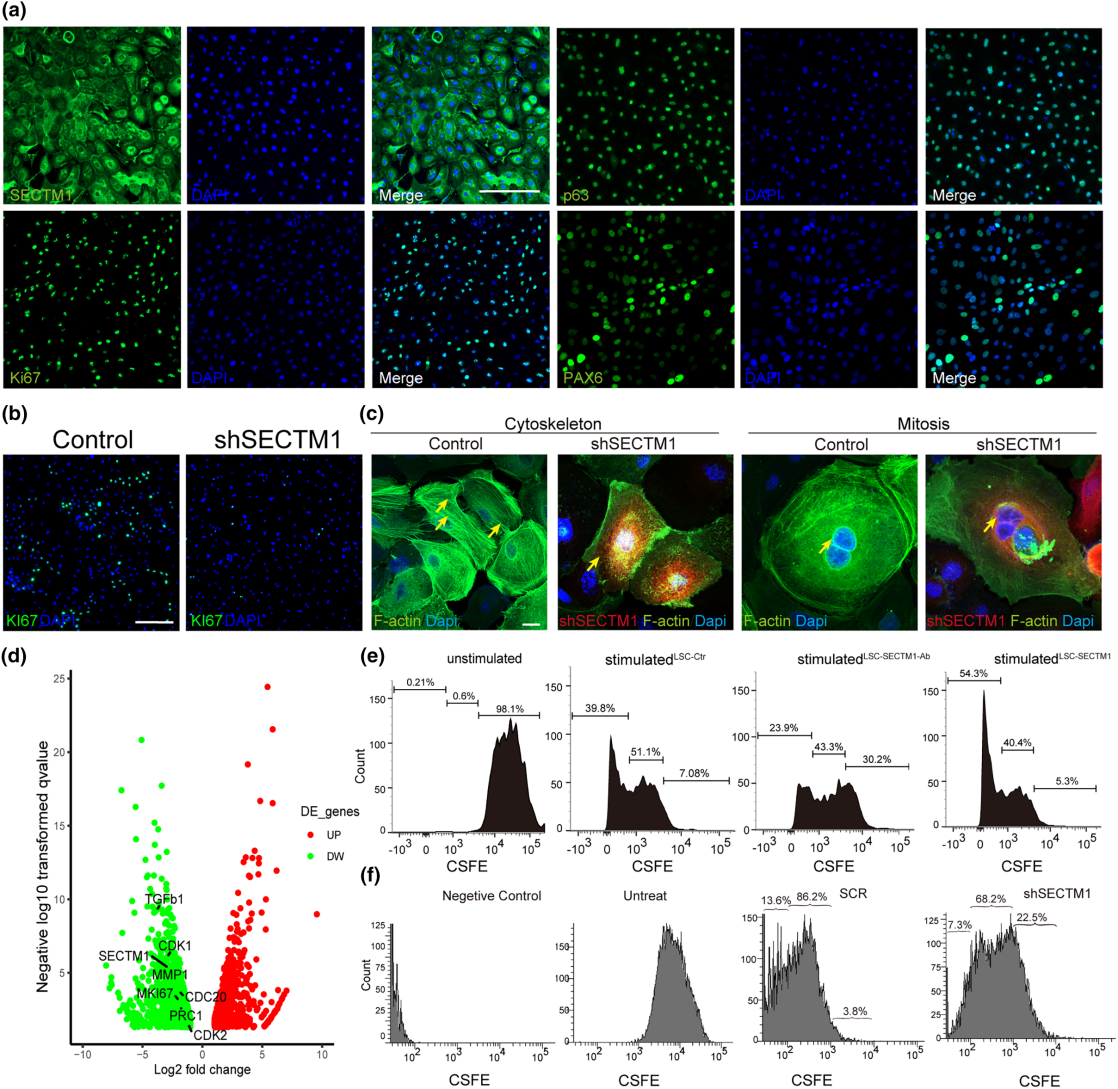
SECTM1 is essential for LSCs proliferation. (a) Immunofluorescent staining of SECTM1, p63, Ki67, and PAX6 of hLSCs. Scalebar = 200um. (b) Immunofluorescent staining of Ki67 in shSECTM1 hLSCs and SCR control. Scalebar = 100um. (c) Confocal imaging of live stained actin cytoskeleton differences in SECTM1 knockdown hLSCs and SCR control. Scalebar = 20um. (d) Volcano plots of shSECTM1 hLSCs differential genes compare with control by threshold of fold change >1, FDR <0.05. (e) CFSE (carboxyfluorescein succinimidyl ester)‐labeled hLSCs were cultured alone or with control IgG, SECTM1 neutralizing antibody or SECTM1 recombinant protein. Proliferation of LSCs was analyzed 4 days later by FACS (Fluorescence activated Cell Sorting). Representative data from three independent experiments are shown. (f) CFSE (carboxyfluorescein succinimidyl ester)‐labeled hLSCs were treated with SCR or shSECTM1 for 1 day and followed by puromycin sieving. Proliferation of LSCs was analyzed 2 days later by FACS (Fluorescence activated Cell Sorting). Representative data from three independent experiments are shown.

We then investigated the role of SECTM1 using primary cultured human LSCs. The LSCs were characterized based on the expression of *Ki67*, *p63*, and *PAX6* (Figure 3a), and SECTM1 expression was confirmed. We also observed a significant reduction in Ki67‐positive cells upon SECTM1 knockdown compared to that of the control group (Figure 3b; Figure S3a,b), suggesting a decrease in proliferation efficiency in shSECTM1‐LSCs. A living‐cell cytoskeleton dye was employed to trace the actin scaffold dynamics during mitosis in the LSCs. We observed disordered actin filaments and nuclear actin nests during mitosis in shSECTM1‐LSCs compared to those in control cells (right panel, Figure 3c). Indeed, the expression of several representative genes associated with cell proliferation and migration were markedly downregulated in shSECTM1‐human Limbal Stem Cells(hLSCs), for example, *Ki67*, *CDK1*, and *MMP1* (Figure 3d; Figure S3c). Meanwhile, no significant difference of ABCG2 and p63α could be found between shSECTM1 and control group, suggesting that SECTM1 may not affect the stemness of LSCs (Figure S3d). These findings suggest a critical role for SECTM1 in LSC replication.

**FIGURE 4:**
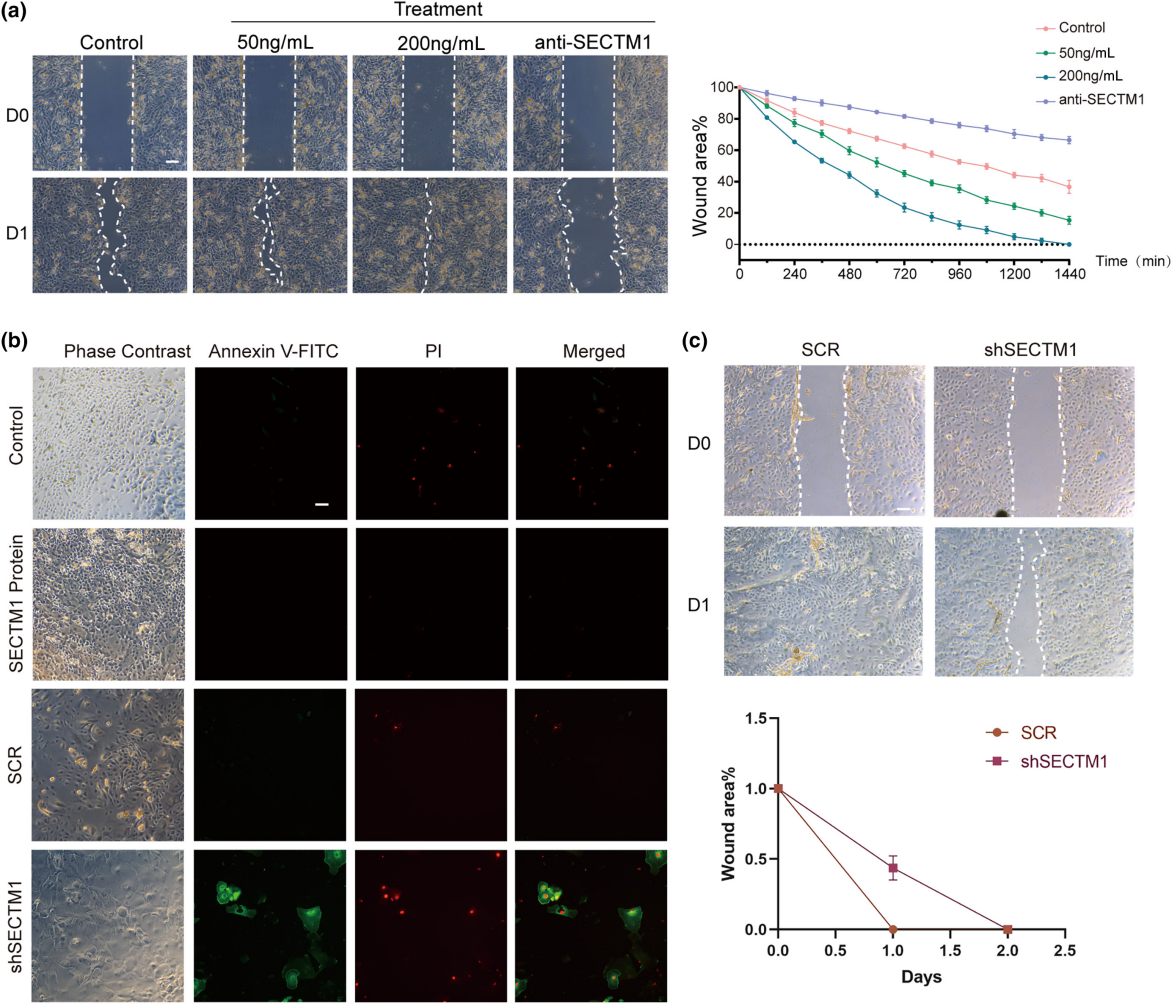
SECTM1 is essential for LSCs migration. (a) Representative phase microscopic images show wound closure at 0 and 24 h. hLSCs were scraped and allowed to heal in medium alone or supplemented with different concentrations of SECTM1 recombinant protein (50 and 200 ng/mL), or with SECTM1 neutralizing antibody. Scalebar = 50um. Quantifications of wound closure area are shown. The remaining acellular areas of hLSCs were photographed and measured after scraping. Data represent means±SD. All experiments were performed in triplicates. (b) Annexin V‐FITC and PI staining of LSCs treated with rbSECTM1 protein, SCR and shSECTM1. Green fluorescence represents Annexin V‐FITC positive staining cells, red fluorescence represents PI positive staining cells. Only stained by green fluorescence is apoptotic cells, double stained by green and red fluorescence cells are necrotic cells. Unstained cells are normal cells. Scalebar = 50um. (c) Representative phase microscopic images show wound closure at 0 and 24 h. hLSCs were transfected with SCR or shSECTM1 iRNA followed by scraped and allowed to heal in medium. Scalebar = 50um. Quantifications of wound closure area are shown. The remaining acellular areas of hLSCs were photographed and measured after scraping. Data represent means±SD. All experiments were performed in triplicates.

To confirm the function of SECTM1, we tested SECTM1‐stimulated or shSECTM1 LSC proliferation using a carboxyfluorescein succinimidyl amino ester (CSFE) staining assay. Flow cytometry results showed that recombinant human SECTM1 (rhSECTM1)‐treated LSCs exhibited stronger cell division (54.3%) than the other treatments: control (39.8%), neutralizing anti‐SECTM1 (23.9%), and unstimulated (0.21%) (Figure 3e). In comparison to the control, shSECTM1 LSCs exhibited reduced cellular division with stronger fluorescent signal (22.5% vs. 3.8%). Meanwhile, 13.6% of the cells in the control group underwent multiple divisions, which is significantly higher than cells in shSECTM1 group (Figure 3f). This finding partially supports the results of an LSC wound‐healing assay. Treatment with rhSECTM1 accelerated LSC proliferation in a dose‐dependent manner, whereas treatment with anti‐SECTM1 significantly inhibited LSC proliferation and migration (Figure 4a). Notably, knockdown SECTM1 resulted in extensive apoptosis and necrosis of LSCs, as confirmed by Annexin V‐FITC/PI staining (Figure 4b). Also, knockdown SECTM1 could limit the migration ability of LSCs (Figure 4c). Taken together, SECTM1 is essential for LSC proliferation and migration.

### 3.4 CDCA7 is a downstream effector of SECTM1

**FIGURE 5:**
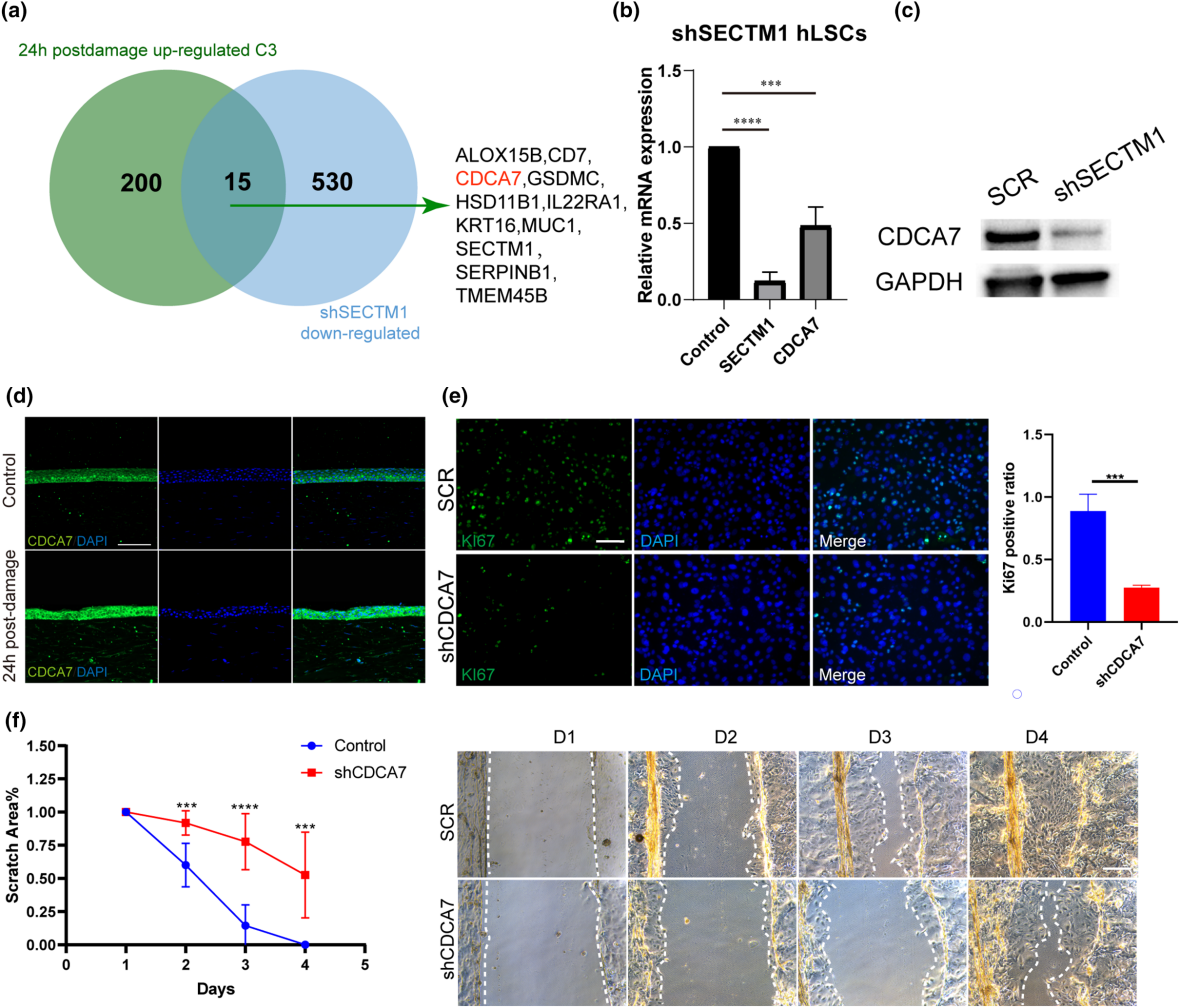
CDCA7 is a downstream gene of SECTM1. (a) Venn diagram exhibits the cross‐section of 24 h post‐damage up‐regulated genes of Figure 2. A C3 cluster and SECTM1 knockdown cell downregulated genes. 15 SECMT1 potential downstream candidates remains. (b) Related expression of *SECTM1* and *CDCA7* in SECTM1 knockdown hLSCs. q‐PCR values were normalized to the values of internal GAPDH. Data are represented as mean ± SD (***p < 0.001, ****p < 0.0001) (c) Western blotting analysis of CDCA7 protein expression in indicated treatment. (d) Immunofluorescent staining of CDCA7 in uninjured and 24 h post‐damage macaques corneas. Scalebar = 100um. (e) Immunofluorescent staining of Ki67 in CDCA7 knockdown LSCs and SCR control. Ki67 positive ratio was represented by mean percentage ± SD. All experiments were performed in triplicates. ***p < 0.001. (f) Representative phase microscopic images show wound closure from Day0 to Day4. CDCA7 knockdown LSCs and SCR control were scraped and allowed to heal in medium. Quantifications of wound closure are shown. The remaining acellular area of hLSCs was photographed measured after scraping. Data represent means ± SD. All experiments were performed in triplicates. ***p < 0.001, ****p < 0.0001.

To explore the molecular mechanism of SECTM1, we narrowed down its potential downstream genes by selecting those at the intersection of the upregulated genes in cluster C3 monkey cornea at 24 h post‐injury and the downregulated genes in shSECTM1‐LSCs (Figure 5a). Most of the 15 candidate genes identified have been shown to participate in cell proliferation, immune response, and cell migration, including *ALOX15B*, *GSDMC*, *CD7*, *IL22RA1*, *KRT16*, *MUC1* and *CDCA7*. CDCA7, a key factor in tumor progression and cell cycle progression, was identified as a promising candidate for further investigation due to its emerging role in cellular processes relevant to our study. qPCR and western blotting revealed a significant decrease in both *CDCA7* mRNA and protein expression levels, in shSECTM1‐LSCs (Figure 5b,c). Immunofluorescence staining of monkey corneas showed that the expression range of CDCA7 shifted from sporadic basal layer cells to widespread expression throughout the corneal epithelium as well as the enhanced expression intensity at 24 h post‐injury (Figure 5d). Similar to the observations in shSECTM1‐hLSCs, shCDCA7‐hLSCs showed a lower percentage of Ki67‐positive cells (Figure 5e), a slower proliferation phenotype (Figure S4a), and a disorganized cytoskeleton during mitosis (Figure S4b). In wound‐healing experiments, disruption of *CDCA7* led to delayed proliferation and migration of LSCs (Figure 5f). Therefore, SECTM1 plays a role in corneal epithelial cell proliferation partially via *CDCA7*.

### 3.5 SECTM1 plays a critical role in corneal wound healing in vivo

**FIGURE 6:**
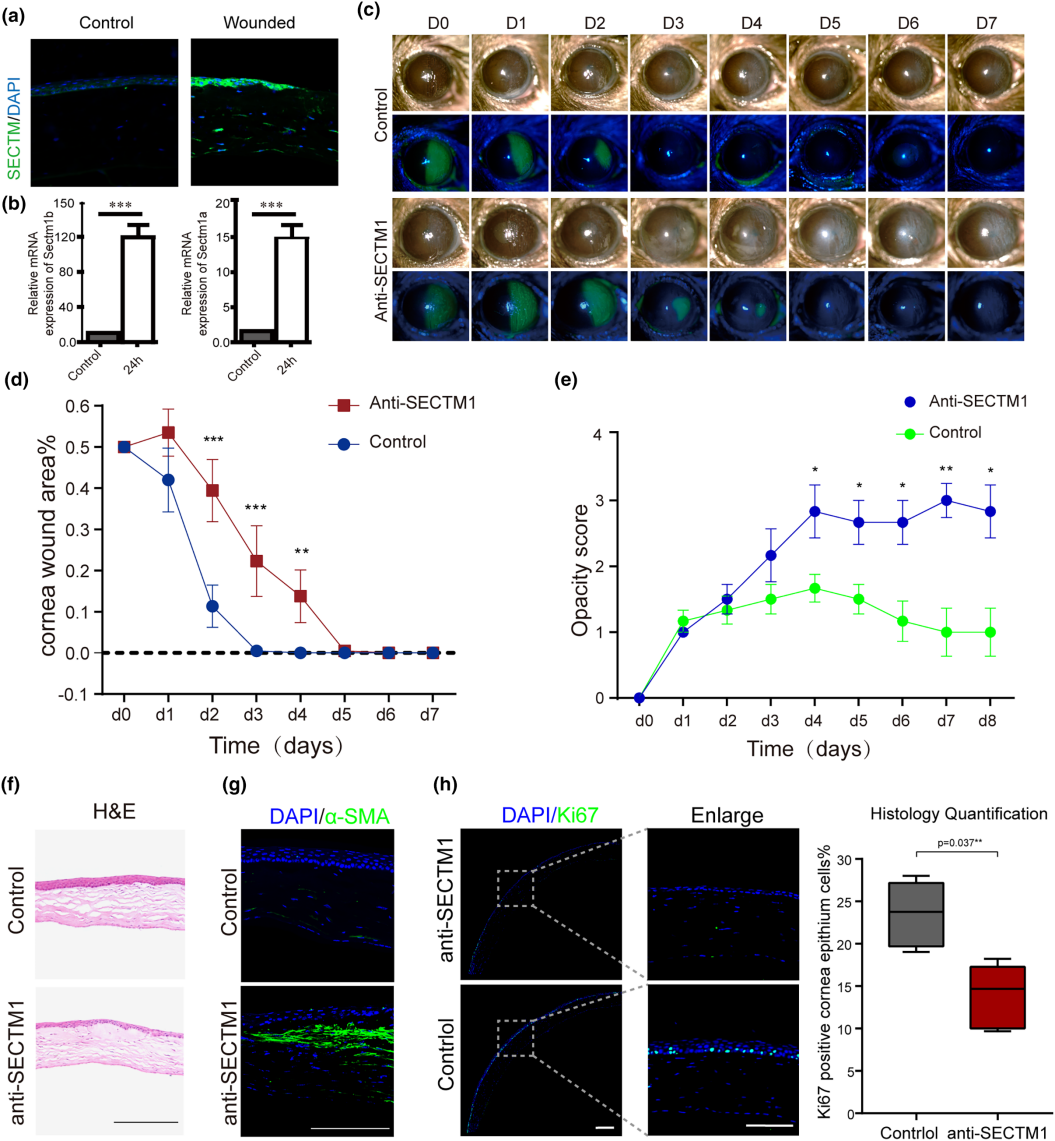
SECTM1 plays a critical role in corneal wound healing in‐vivo. (a) Immunofluorescent staining of SECTM1 in uninjured and 24 h post‐damage mouse corneas. Scalebar = 100um. (b) Related mRNA expression level of 24 h post damage of SECTM1 in mouse corneal wound edge. q‐PCR values were normalized to the values of internal GAPDH. Data are represented as mean ± SD. (c) Representative images of the temporal re‐epithelization process that occurs following cornea wounding (*t* = 0) in control and SECTM1 blocking antibody treated mice eyes. Slit‐lamp photographs of wounded cornea at each time point (d0, d1, d2, …d7 after the scrape of corneal epithelium). Fluorescein green staining was used to identify the area of epithelial defect. (d) Quantification of re‐epithelialization in control and SECTM1 blocking antibody treated wounds. Epithelial defect is presented as the percentage of the original wound size. Students *t*‐test was used to measure statistical significance, *n* = 5. Data are represented as mean ± SD (**p < 0.01, ***p < 0.001, compared with the control group with the isotype antibody injection). (e) Corneal opacity is evaluated by opacity score. Kruskal–Wallis test was used to measure statistical significance, *n* = 6. Data are represented as mean ± SEM (*p < 0.05, **p < 0.01, compared with control group) (f) HE staining of SECTM1 antibody treated healed mouse corneas, scalebar =200um. (g) Immunofluorescent staining of alpha‐SMA antibody treated healed mouse corneas, scalebar =200um. (h) Immunofluorescent stating of Ki67 on mice cornea treated with SECTM1 antibody post damage and the quantification of its positive ratio, Data are represented as mean ± SD.

Loss‐of‐function experiments were performed on injured mouse corneas to evaluate the function of SECTM1 in vivo. SECTM1 expression was upregulated at 24 h post‐wounding (Figure 6a,b). Neutralization of SECTM1 expression significantly delayed wound closure by 2 days (Figure 6c,d). Ki67 expression was markedly blocked by the anti‐SECTM1 antibody in the wounded corneal epithelium (Figure 6h).

It should be noted that the quality of repaired vision was unsatisfactory when the injured cornea was treated with anti‐SECTM1. Along with corneal opacity, the wounded mouse corneas showed disorganized stroma and thinner layers of epithelium in the anti‐SECTM1 group than in the control group (Figure 6e,f). This phenotype was further confirmed by the staining of alpha‐smooth muscle actin (α‐SMA), a well‐known marker for fibroblasts. α‐SMA‐positive cells could only be detected in anti‐SECTM1‐treated cornea, indicating the development of corneal fibrosis when SECTM1 was interrupted during wound healing (Figure 6g). Therefore, SECTM1 is essential for corneal repair. The absence of SECTM1 delays corneal wound closure and alters corneal transparency.

### 3.6 SECTM1 rescues wound repair in aged cornea

Owing to the crucial role of SECTM1 in corneal repair and its deficiency in aged corneas, we assessed whether the potential enhancement of SECTM1 expression would improve aged corneal wound healing. When SECTM1 protein was applied to injured corneas of aged monkeys and mice.

**FIGURE 7:**
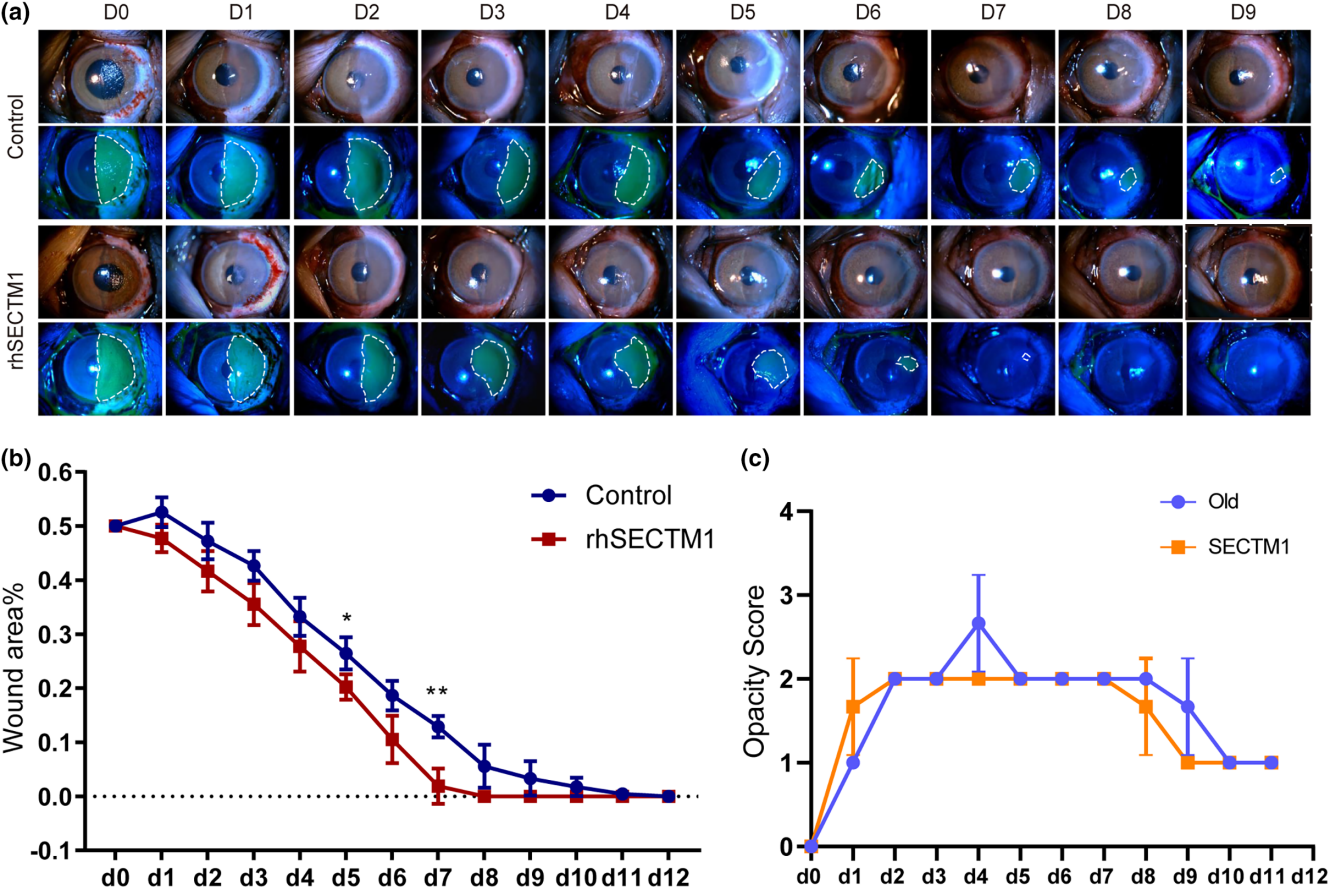
SECTM1 rescues wound repair in aged cornea. (a) Representative images of the temporal re‐epithelization process that occurs following cornea wounding (*t* = 0) in aged control and SECTM1‐treated macaque eyes. Slit‐lamp photographs of aged control and SECTM1‐treated macaques wounded cornea at each time point (d0, d1, d2…, d9 after the scrape of corneal epithelium). Green‐colored areas represent fluorescein‐stained regions of the corneal epithelial wounds. (b) Quantification of re‐epithelialization in control and rhSECTM1‐treated wounds. Epithelial defect is presented as the percentage of the original wound size. Students *t*‐test was used to measure statistical significance, *n* = 3. Data are represented as mean ± SD (*p < 0.05, **p < 0.01, compared with the control group). (c) Corneal opacity is evaluated by opacity score. Kruskal–Wallis test was used to measure statistical significance, *n* ≥ 3. Data are represented as mean ± SEM.

The monkey corneas healed at 12 days in the control group, whereas topical treatment with SECTM1 accelerated wound closure by 7 days (Figure 7a,b). However, no improvement in corneal opacity was observed after SECTM1 treatment until the end of the healing process (Figure 7c). Strikingly, the addition of SECTM1 not only accelerated mouse corneal wound closure (SECTM1 group: 4 days vs. control group: 6 days) (Figure S5a,b) but also improved corneal transparency after recovery (Figure S5c). Taken together, external supplementation with SECTM1 replenished the lack of SECTM1 protein in aged corneas, improving healing.

## 4 DISSCUSION

Aging is an intrinsic process that is accompanied by a decrease in or loss of a series of functions that influence organ homeostasis (Parrish, 2017). Here, we found that wound healing in aged primate corneas was delayed, and repair quality was poor. Mechanistically, the wounded corneal epithelium in the aged monkeys failed to initiate the expression of SECTM1, a novel cytokine critical for corneal recovery. Treatment with SECTM1 improved the corneal healing process in monkeys and mice and thus holds great promise for clinical applications.

Tissue wound healing is a complex biological process that involves inflammation, cell migration, and proliferation (Pugliese et al., 2018). Poor wound healing in aged people has been well‐documented in many organs and tissues (Thanapaul et al., 2021). Aged keratinocytes display reduced proliferation and migration due to impaired communication with immune cells (Keyes et al., 2016). In this study, the delayed corneal wound healing observed with aging may be attributed to the hindered or delayed activation of adaptive cell proliferation at the transcriptional level. A plausible inference is that different injury‐response gene sets may influence the subsequent repair strategies in the cornea. Stronger and sustained upregulation of expression of cell proliferation‐related genes in the young cornea promoted faster repair, whereas dramatic cell‐matrix adhesion‐related genes up‐regulation was observed in the aged cornea. Despite age‐related structural and morphological changes in the corneal epithelium, we found no significant difference in the transcriptional profile of corneal epithelial tissues between young and aged monkeys in a homeostatic state. This finding is consistent with other studies in mice. For example, aged mice maintain an epidermis that is transcriptionally similar to that of young mice without injury; only mild changes in gene regulation can be found during homeostatic renewal of LSCs (Lin et al., 2023).

SECTM1 is a type I transmembrane glycoprotein localized in the Golgi apparatus and exists in a transmembrane and soluble form (Slentz‐Kesler et al., 1998). Several studies have reported that SECTM1, an immunoregulatory factor, stimulates neutrophil proliferation (Kamata & Yamamoto, 2016); however, no corneal function has been reported to date. It would be interesting to test whether SECTM1 facilitates regeneration in other epithelial tissues. Interestingly, there are a number of known cytokines and growth factors that also exist in both transmembrane and secreted forms, for example, TNFα (Horiuchi et al., 2010) and TGFβ (Yang et al., 2013). Since treatment with recombinant SECTM1 can stimulate LSC growth and corneal wound healing, it is plausible that SECTM1 regulates cell proliferation through its extracellular soluble form. Therefore, it is necessary to study how SECTM1 fulfills this function. Although our data suggest that CDCA7 (a gene that regulates the cell cycle) acts downstream of SECTM1, the underlying mechanism remains to be explored.

## AUTHOR CONTRIBUTIONS

H.O. provided the idea, conceived this project and contributed to writing and discussion. J.Z., X.L., and Q.K. designed and preformed the experiment. J.Z. preformed the data analysis and wrote the manuscript. K.M. performed animal experiments. W.Z., Y.H., J.T., L.W., J.P.J., helped the experiments.

## CONFLICT OF INTEREST STATEMENT

The authors declare no competing interests.

## Supporting information


Figure S1.

Table S1.

## Data Availability

All relevant data supporting the key findings of this study are available within the article and its supplementary Information files or from the corresponding author upon reasonable request.
