# Supplementary material for: Deficiency of SECTM1 impairs corneal wound healing in aging
Source: Aging Cell. 2024 Jun 17;23(10):e14247. doi: 10.1111/acel.14247 (PMC11464118; doi:10.1111/acel.14247)
Supplement: Supplementary file 1 — Figure S1. [file ACEL-23-e14247-s002.docx]

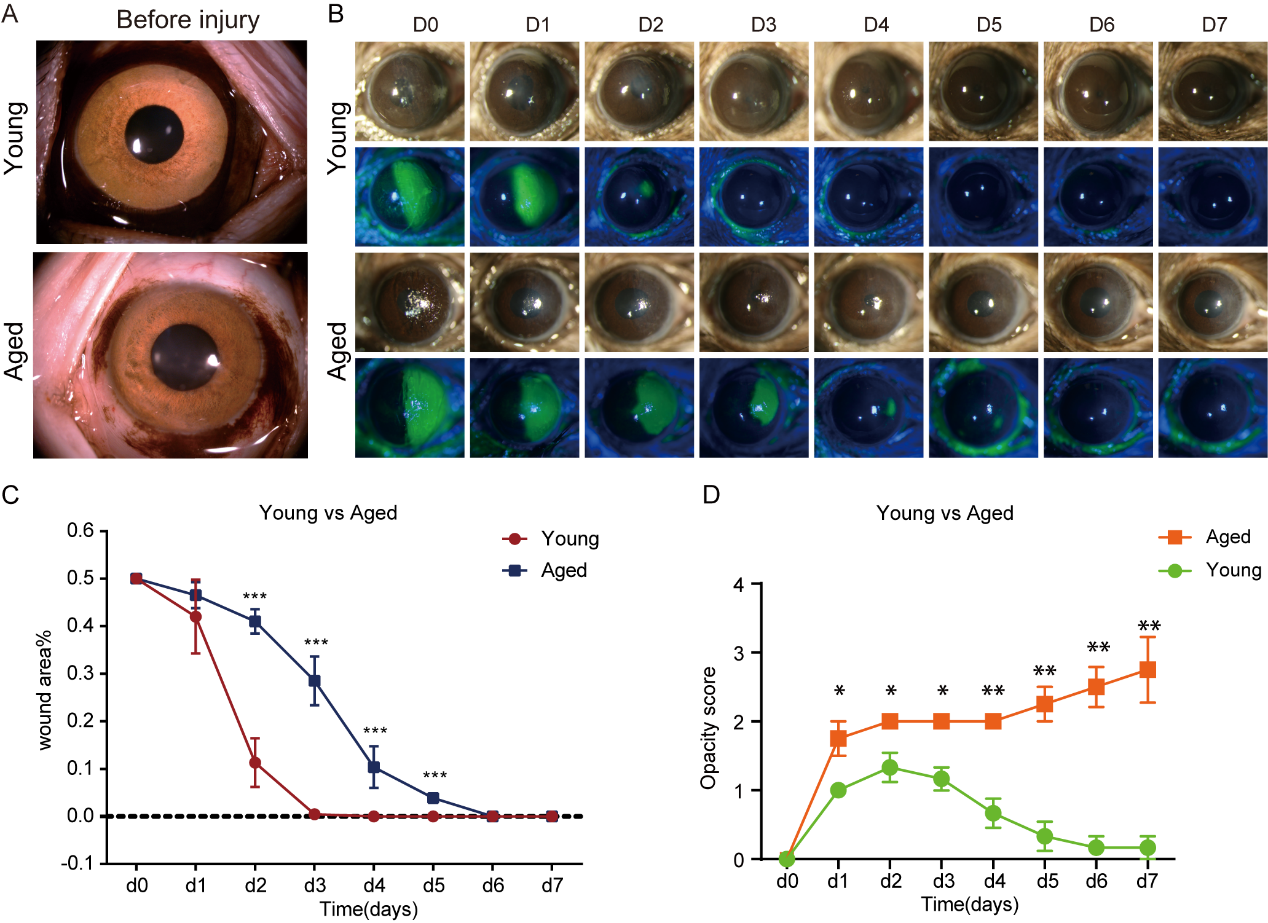


**Figure1S Corneal wound healing process in young and aged mice.**

1. Slit-lamp photographs of young and aged macaque cornea before injury.
2. Slit-lamp photographs of young and aged mouse wounded corneas at each time point (d0,d1,d2,...,d7 after the scrape of corneal epithelium). Fluorescence sodium dyeing was used to identify the area of epithelial defect.
3. Quantification of re-epithelialization in young and aged wounds. Epithelial defect is presented as the percentage of the original wound size. Students t test was used to measure statistical significance, n = 3. Data are represented as mean ± SD (**P* < 0.05, ***P* < 0.01, ****P* < 0.001 , compared with young group).
4. Corneal opacity is measured by opacity score. Kruskal-Wallis test was used to measure statistical significance, n ≥ 4. Data are represented as mean ± SEM (**P* < 0.05, ***P* < 0.01, compared with young group).


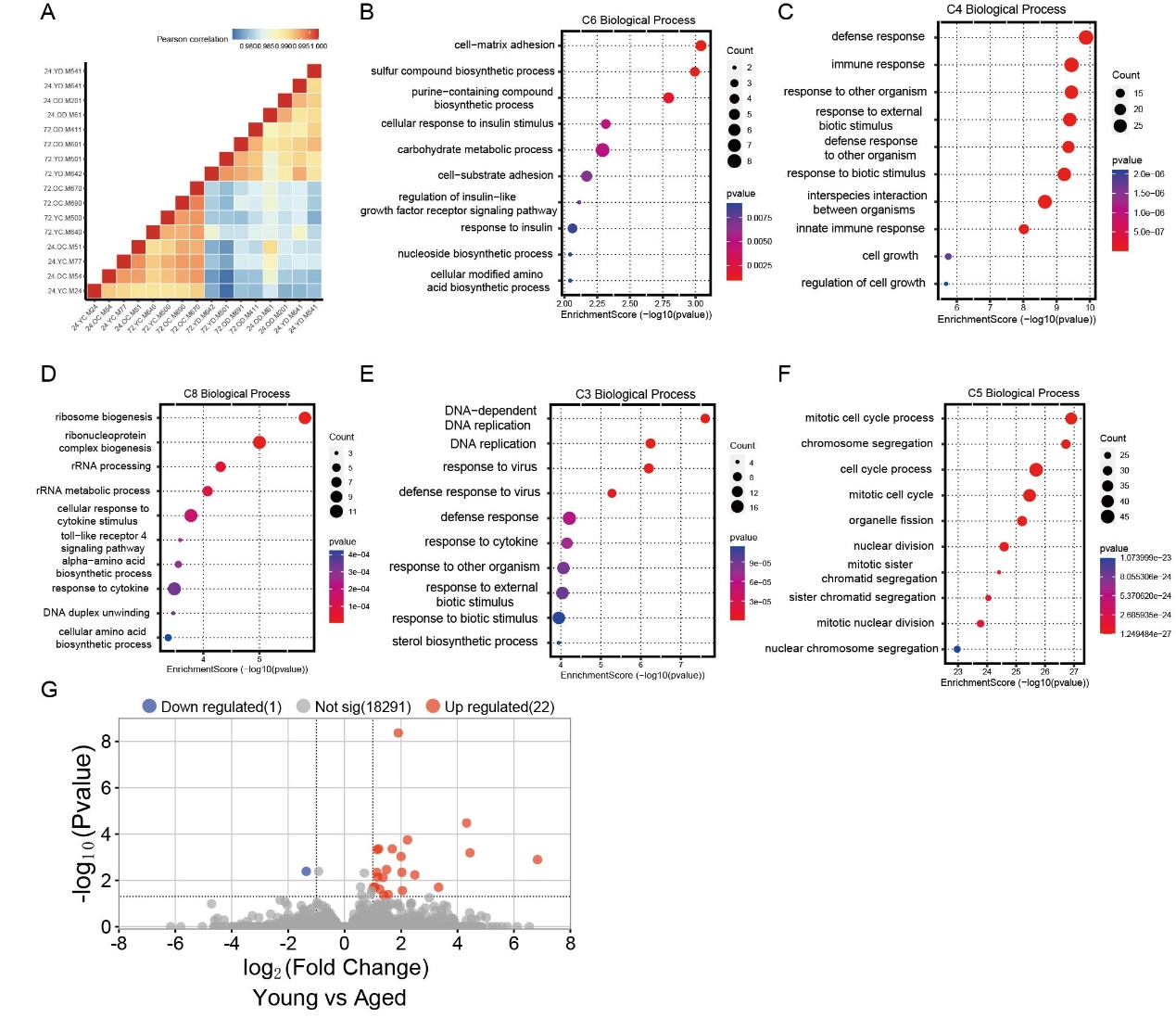


**Figure 2S Transcriptional differences in young and aged macaque corneas before and after injury.**

1. Pearson correlation between all analyzed samples. Sample naming rule: 24=24h post damage; 72 = 72h post damage; YC = young control; OC = aged control; YD = young post damage; OD = aged post damage; MXXX= macaques code.
2. –(F) different clusters GO BP analysis.

(G) Volcano plots of young and aged cornea RNA-seq DEseq2 analysis. Orange points represent genes that are expressed as upregulation fold change > 1 and blue represent as downregulation fold change > 1, FDR is < 0.05. Total differential genes constitute 0.125% (23/18314) of total genes.


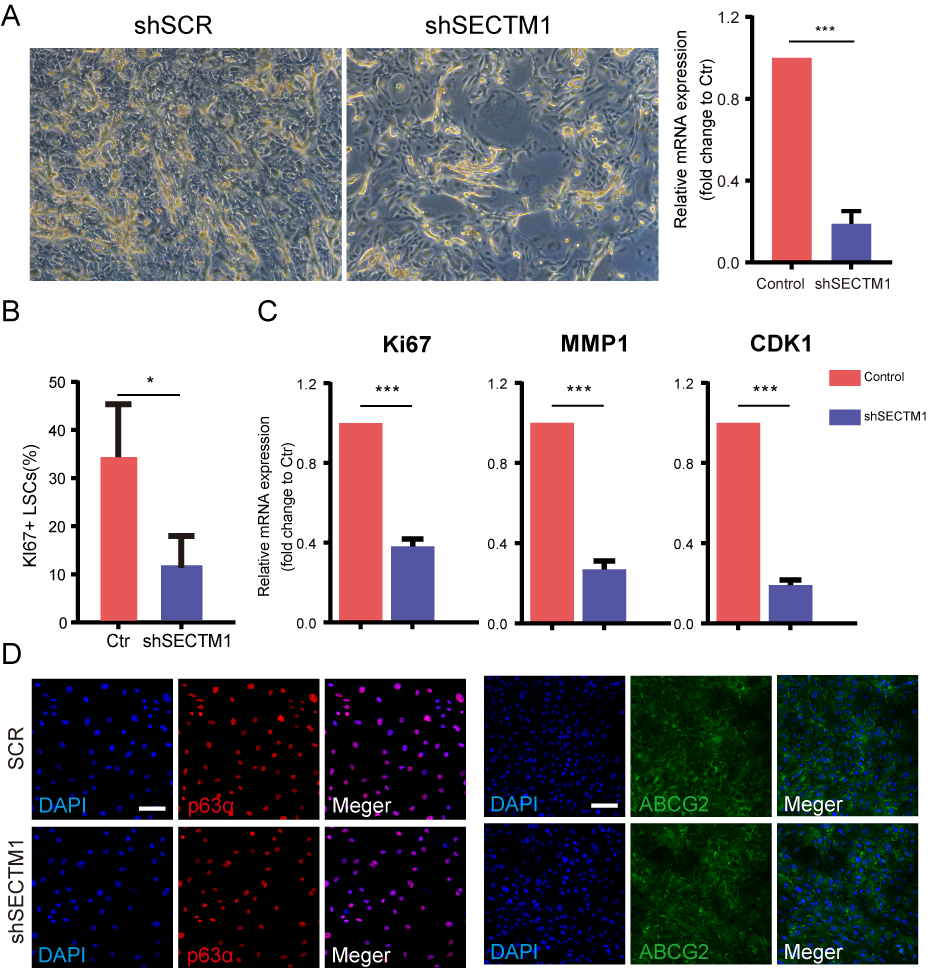


**Figure 3S SECTM1 affects numerous cell proliferation-related markers expression in hLSCs.**

1. Morphological changes in LSCs upon SECTM1 knockdown. q-PCR showed decreased expression in SECTM1 knockdown LSCs. q-PCR values were normalized to the values of internal GAPDH. Scalebar=100um.
2. Quantification of Ki67 positive ratio between SECTM1 knockdown LSCs and SCR control. Data represent means±SD. All experiments were performed in triplicates. **P* < 0.05
3. Q-PCR results showed Ki67, MMP1, CDK1 mRNA expression were decreased in SECTM1 knockdown LSCs by different levels. q-PCR values were normalized to the values of internal GAPDH. Data represent means±SD, ****P* < 0.001.
4. Immunofluorescent staining of p63α and ABCG2 staining in shSECTM1 and SCR LSCs control. Scalebar = 50um.


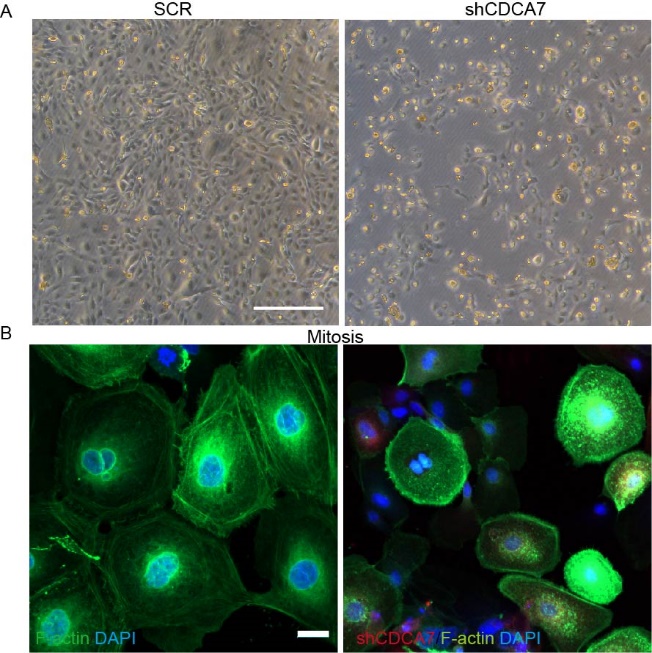


**Figure 4S CDCA7 knockdown affects hLSCs morphology.**

1. Morphological changes in LSCs upon CDCA7 knockdown. Scalebar=100um.
2. Confocal imaging of live-stained cytoskeleton in CDCA7 knockdown LSCs and SCR control during mitosis. Scalebar = 20um


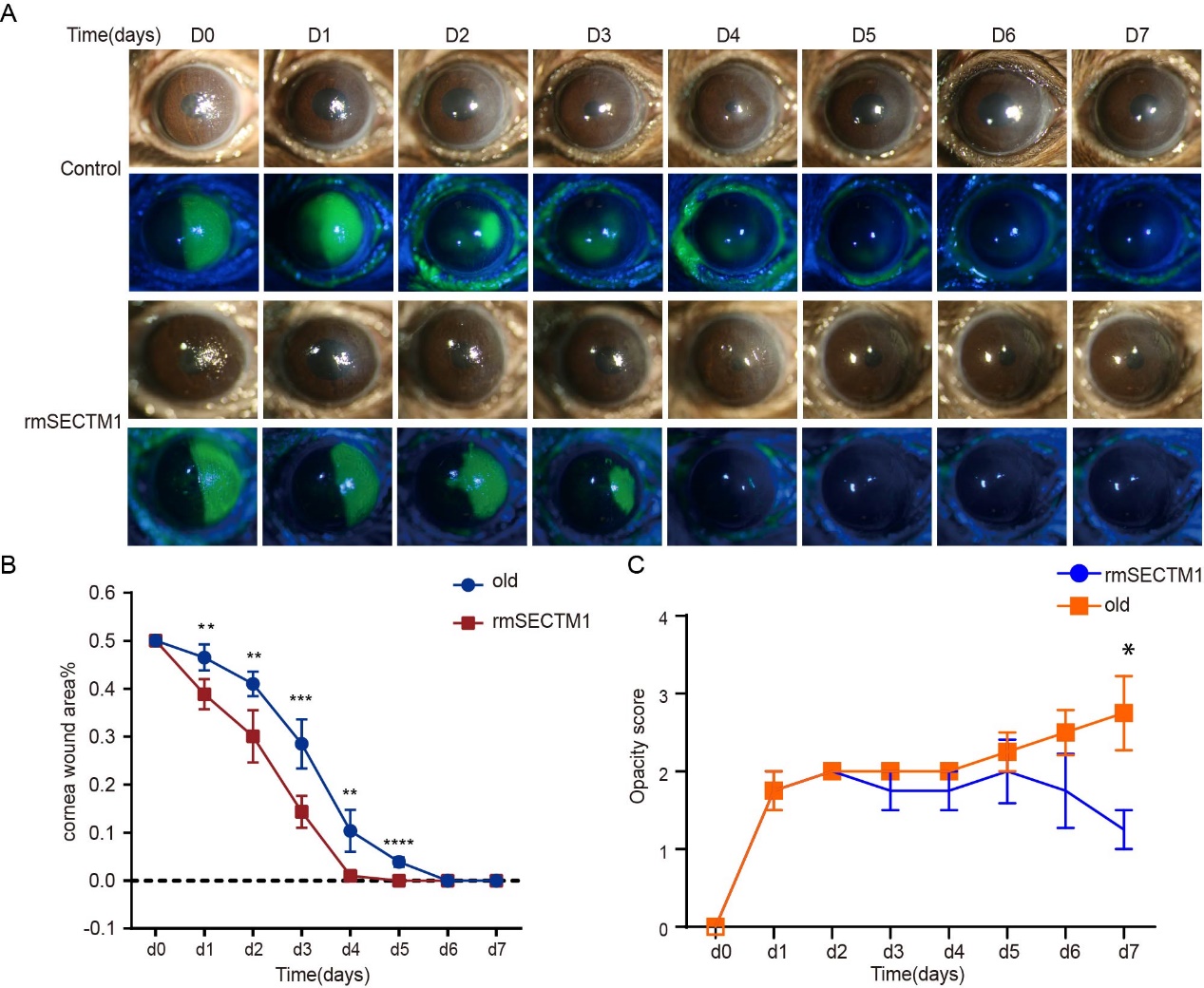


**Figure 5S SECTM1 rescues wound repair in aged mouse cornea.**

1. Representative images of the temporal re-epithelization process that occurs following cornea wounding(t=0) in aged control and SECTM1-treated mouse eyes. Slit-lamp photographs of aged control and SECTM1-treated mouse wounded corneas at each time point (d0, d1, d2…d9 after the scrape of corneal epithelium). Green-colored areas represent fluorescein-stained regions of the corneal epithelial wounds.
2. Quantification of re-epithelialization in control and rmSECTM1-treated wounds. Epithelial defect is presented as the percentage of the original wound size. Students t test was used to measure statistical significance, n = 3. Data are represented as mean±SD (***P* < 0.01, ****P* < 0.001, *****P* < 0.0001, compared with the control group).
3. Corneal opacity is measured by opacity score. Kruskal-Wallis test was used to measure statistical significance, n ≥ 4. Data are represented as mean ± SEM (**P* < 0.05, compared with young group).
