# Supplementary material for: Deficiency of SECTM1 impairs corneal wound healing in aging
Source: Aging Cell. 2024 Jun 17;23(10):e14247. doi: 10.1111/acel.14247 (PMC11464118; doi:10.1111/acel.14247)
Supplement: Supplementary file 2 — Table S1. [file ACEL-23-e14247-s001.docx]

Supporting information

Table S1. Antibody List

| Antibody | Brand | Catlog # |
| --- | --- | --- |
| SECTM1 | Proteintech | #60281-1-lg |
| CDCA7 | Proteintech | #15249-1-AP |
| PAX6 | Sigma | #AMAB91372 |
| P63 | CST | #67825S |
| Ki67 | CST | #9129S |
| Alpha-SMA | thermofisher | #MA1-06110 |
| Alexa Fluor 488-labeled donkey anti-mouse | CST | #4408 |
| Alexa Fluor 488-labeled donkey anti-rabbit IgG | thermofisher | #A11008 |

Table S2. The list of primers of the genes for qRT-PCR

| Gene name | Forward Primer | Reverse Primer |
| --- | --- | --- |

| MKI67 | GCCTGCTCGACCCTACAGA | GCCTGCTCGACCCTACAGA |
| --- | --- | --- |
| MMP1 | TCTGACGTTGATCCCAGAGAGCAG | CAGGGTGACACCAGTGACTGCAC |
| GAPDH | TGTTGCCATCAATGACCCCTT | CTCCACGACGTACTCAGCG |
| SECTM1 | CGCCATCTTCAATGAGGTGG | CCAGCGTGACTTGTCTGTTATT |
| CDK1 | AAACTACAGGTCAAGTGGTAGCC | TCCTGCATAAGCACATCCTGA |
| CDCA7 | GGGTGGCGATGAAGTTTCCA | GGGGATGTCTTCCACGGAAC |
| mGAPDH | TGACCTCAACTACATGGTCTACA | CTTCCCATTCTCGGCCTTG |
| mSectm1a | TCAGTGCCTGCTATCCCTACC | GGGGCTTTTTATCGAAGATGGT |
| mSectm1b | TTCCCCTGGTCACTGTCAGC | CACAGCTTGTAGGTGGCACAC |
